# Endoscopic optical coherence tomography angiography using a forward imaging piezo scanner probe

**DOI:** 10.1002/jbio.201800382

**Published:** 2019-01-30

**Authors:** Lara M. Wurster, Ronak N. Shah, Fabian Placzek, Simon Kretschmer, Michael Niederleithner, Laurin Ginner, Jason Ensher, Michael P. Minneman, Erich E. Hoover, Hans Zappe, Wolfgang Drexler, Rainer A. Leitgeb, Çağlar Ataman

**Affiliations:** ^1^ Center for Medical Physics and Biomedical Engineering Medical University of Vienna Vienna Austria; ^2^ Christian Doppler Laboratory for Innovative Optical Imaging and its Translation to Medicine Medical University of Vienna Vienna Austria; ^3^ Gisela and Erwin Sick Chair of Micro‐optics, Department of Microsystems Engineering University of Freiburg Freiburg Germany; ^4^ INSIGHT Photonics Solution, Inc. Lafayette Colorado

**Keywords:** angiography, endoscopy, MEMS technology, optical coherence tomography

## Abstract

A forward imaging endoscope for optical coherence tomography angiography (OCTA) featuring a piezoelectric fiber scanner is presented. Imaging is performed with an optical coherence tomography (OCT) system incorporating an akinetic light source with a center wavelength of 1300 nm, bandwidth of 90 nm and A‐line rate of 173 kHz. The endoscope operates in contact mode to avoid motion artifacts, in particular, beneficial for OCTA measurements, and achieves a transversal resolution of 12 μm in air at a rigid probe size of 4 mm in diameter and 11.3 mm in length. A spiral scan pattern is generated at a scanning frequency of 360 Hz to sample a maximum field of view of 1.3 mm. OCT images of a human finger as well as visualization of microvasculature of the human palm are presented both in two and three dimensions. The combination of morphological tissue contrast with qualitative dynamic blood flow information within this endoscopic imaging approach potentially enables improved early diagnostic capabilities of internal organs for diseases such as bladder cancer.

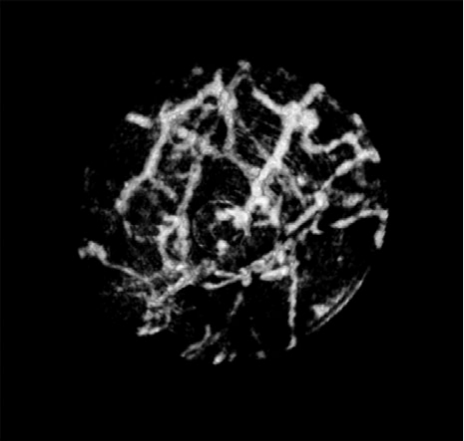

## Graphical abstract

A forward imaging endoscope providing morphological information by optical coherence tomography (OCT) as well as visualization of microvasculature by optical coherence tomography angiography (OCTA) has the potential to improve diagnosis of diseases such as bladder cancer at an early stage. In this paper, an endoscope of such kind is presented and its potential is demonstrated by presenting OCT images of the finger and OCTA images of the palm.

## 1 INTRODUCTION

Optical coherence tomography (OCT) is a non‐invasive, cross‐sectional, high**‐**resolution optical imaging modality. By taking advantage of the optical transparency of the human eye, the technology found its most successful medical application in ophthalmology for providing histology‐like retinal tomograms 1, 2. In other tissues of the human body, however, OCT's penetration depth is severely limited to a few millimeters by increased scattering and absorption characteristics. Thus, to enable assessment of target tissues within the body endoscopic OCT was developed, allowing for non‐invasive imaging of inner organs such as the gastrointestinal tract, the coronary vasculature or the bladder 3, 4, 5, 6, 9. There, the main challenge is the realization of a scanning mechanism within the endoscope probe head to allow three‐dimensional **(**3D**)** image acquisition without significantly increasing the size of the instrument. The integration of an OCT channel in current white‐light endoscopes would complement superficial reflectance imaging with depth information. Besides the purely 3D morphological image contrast in conventional OCT, simultaneously obtained functional contrast may be exploited to visualize movement of particles such as blood cells in the vasculature. This label**‐**free alternative to fluorescein angiography for in vivo delineation of vessel networks is often referred to as optical coherence tomography**‐**based angiography (OCTA) 7, 8. The combination of static morphological imaging with dynamic tissue contrast in endoscopic OCTA, might assist in the early diagnosis of diseases, such as bladder cancer 5, 6.

The very first OCT endoscopes aimed to image tubular organs and are thus labeled as side‐imaging probes 3. As of today these endoscopes are still the most established ones being already introduced commercially into the clinics, for example, in cardiology and gastroenterology. In such an endoscope, the distal probe optics are either rotated from the proximal end by an optical rotary junction or by a small motor in the distal probe head 4, 9. By rotation of the probe optics, including a reflective element (eg, prism or mirror) to direct the light beam to the side, and axial translation of the endoscope, a 3D volume can be acquired. For hollow organ imaging (eg bladder, stomach, nasal cavity, etc.), or for image‐guided surgery, however, the tissue of interest is located in front of the probe and a forward‐imaging OCT endoscope is required. Therefore, more sophisticated scanning mechanisms are needed 10. Furthermore, to facilitate its use as the rigid end of a flexible endoscope, the probe length should be kept at a minimum. Microelectromechanical systems (MEMS) technology offers the most promising approach in this regard. While MEMS‐based mirrors add size, electrothermal MEMS‐based actuation of a fiber requires a long rigid length of the endoscope 5, 11. State‐of‐the‐art solutions incorporate a small piezoelectric tube to scan a fiber in a Lissajous or spiral pattern. This piezoelectric scanning approach has been developed not only for OCT 12 but also for various other imaging modalities mainly by the groups of Li 13 and Seibel 14. The smallest piezo endoscope head with a diameter of 1.6 mm and rigid length of 13.5 mm for OCT has recently been reported. However, this probe has a relatively small field of view (FOV) of 800 μm 15. First endoscopic OCTA images were also recently demonstrated using a larger probe over a FOV of only 450 μm 16. No 3D images have been demonstrated and the technique was not sensitive enough to detect small vessels due to a strong noise background present in the images 16.

In this paper, we report a forward imaging piezo endoscope head design for in vivo OCT and OCTA imaging with a short rigid head length of 11.3 mm. It operates in contact mode to limit motion artifacts and achieves a FOV of up to 1.3 mm in diameter. We present morphological and functional dermatologic imaging results of a human palm and fingertip.

## 2 METHODS

**Figure 1:**
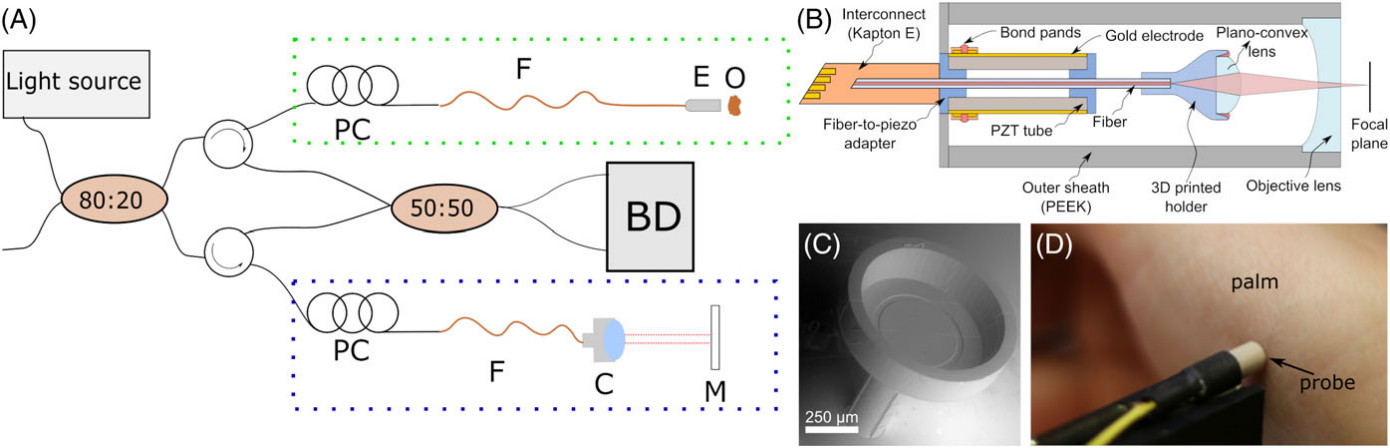
(A) Schematic overview of the OCT setup, reference/sample arm indicated by blue/green dotted squares, respectively, PC, polarization control paddles; F, single mode fiber; C, collimator; M, mirror; E, endoscope head; S, sample; BD, balanced detection unit. (B) Schematic cross‐sectional depiction of the rigid probe head. The fiber cantilever is scanned in a spiral pattern via the piezoelectric tubular actuator (PI Ceramic GmbH—PIC 151, length 3.7 mm, outer diameter 0.8 mm, wall thickness 0.15 mm). A custom‐developed, 10‐μm thick polyimide cable is used to electrically access the four electrodes of the piezo‐tube. For details, please see 19. (C) SEM image of the 3D printed fiber‐to‐lens assembly part (weight with lens: 1.96 mg). (D) Measurement procedure with palm placed in contact with the probe

**Figure 2:**
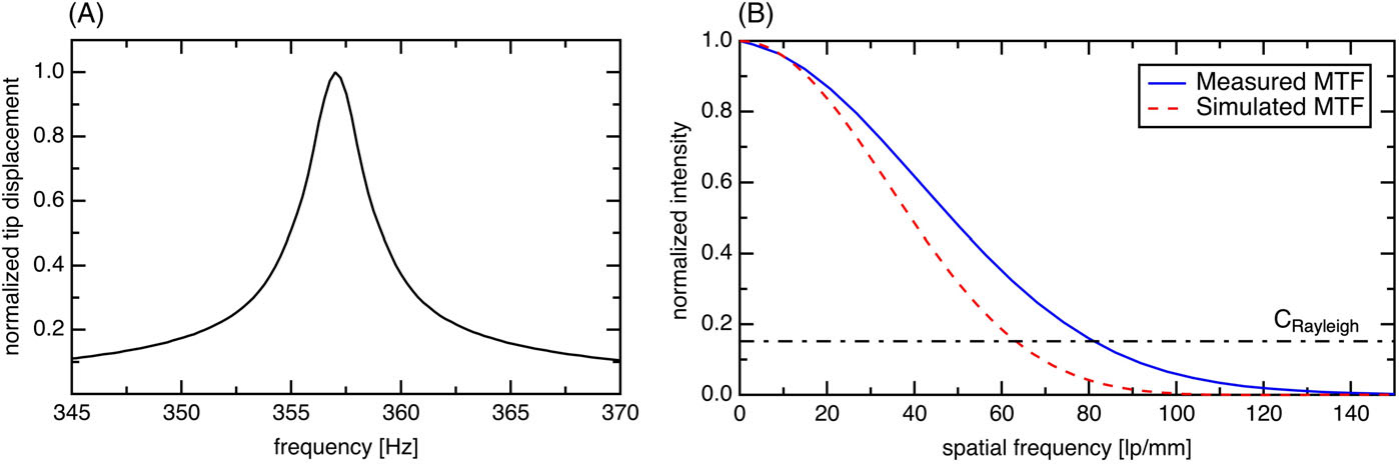
Optomechanical characterization results for the OCT probe. (A) The frequency response measured using a position sensitive detector. (B) Modulation transfer function of the probe measured via the edge spread function. The measured resolution is 12 μm in air, according to the Rayleigh criteria

An akinetic tunable light source (Insight Photonic Solutions, Inc., Lafayette, Colorado) with a center wavelength of 1300 nm, bandwidth of 90 nm and a sweep repetition rate of ~174 kHz has been used for the experiments 17. The output power of the source was ~42 mW and using the OCT setup configuration depicted in Figure 1A an incident power on the sample in front of the endoscope of ~14 mW was achieved. More information on the imaging setup previously developed by the authors can be found elsewhere 18. To compensate for the additional length and dispersion introduced by the endoscope, the same fiber was also added to the reference arm. The OCT system featured an axial resolution of 12 μm and achieved a sensitivity of 104 dB without the endoscope. However, due to increased back reflection, residual dispersion mismatch, and a mismatch of the numerical aperture (NA)–between the OCT systems' fiber (SMF28, NA 0.14) and the fiber of the endoscope (Thorlabs Inc., Newton, New Jersey, SM980G80, NA 0.18)–the original sensitivity was reduced to 96 dB in the final endoscopic OCT setup. Scanning is attained by addressing the four radial electrodes of the piezo‐tube with sinusoidal signals of the same frequency but different phase and amplitude, such that the fiber tip follows a circular pattern. By continuously scaling the amplitude of the driving signals, a spiral scan pattern is obtained. To amplify the piezo displacement, the actuation frequency has to be close to the resonance frequency of the fiber 19, 20. Figure 1 B,D present a schematic depiction and image of the endoscopic probe head, respectively. At the tip of the fiber, the fiber bears a 3D printed holder (Nanoscribe GmbH Photonic Professional GT, Figure 1C) that accommodates a plano‐convex lens (Edmund Optics, #45–966, 1 mm diameter, 0.6 mm focal length). The holder is designed to maintain a 531 μm distance between fiber tip and lens to provide a magnification of 4.29. At the distal end, a plano‐concave lens (Edmund Optics, #67**‐**979, 3 mm diameter, −6 mm focal length) is placed to provide both tip‐encapsulation and FOV expansion. All components of the rigid endoscope head are assembled within a polyether ether ketone tube of 3 mm inner and 4 mm outer diameter. This scanner is adapted from the Fourier‐plane scanner recently developed by the authors 19 with reduced length and enhanced transverse resolution and FOV: the key difference being the off‐the‐shelf focusing plano‐convex lens instead of a collimating GRIN lens. This arrangement provides the same refractive power at a significantly shorter length. Reducing the axial extent of the tip weight also improves the optical performance of the cantilever by enhancing the tip rotation angle at a fixed displacement. Moreover, with a converging beam leaving the scanning lens, the need for an objective lens is eliminated, leading not only to a more compact packaging, but also a potentially larger FOV than the probe diameter of maximal 1.6 mm (given sufficient working distance and scan angle is provided). Figure 2A plots the experimental frequency response of the fiber scanner. The resonant frequency is 358 Hz and the assembly precision is indicated by the symmetry of the curve. During measurements, the best results regarding FOV and amplitude stability were obtained at an actuation frequency of 360 Hz. Optically, the modulation transfer function (plotted in Figure 2B) demonstrates that at 15% contrast, the probe has a resolution of 12 μm in air, exceeding the theoretical expectation by 20%. This is attributed to a deviation in the axial position of the plano‐convex lens in relation to the fiber facet. This interpretation is consistent with the measured depth of field (DOF) of 677 μm, instead of 1 mm, due to the increased NA.

OCT data was recorded at a volume rate of 0.5 Hz. However, data acquisition took only place during the growing spiral pattern, such as a whole C‐scan (containing 360 concentric tomograms of different radii, average pitch between them: ~1.6 μm) was acquired in 1 second. Before each measurement session, a position calibration of the piezo scanner was performed. For this task, the scan pattern was imaged onto a position sensor (Spotana‐9L, Duma Optronics, Israel). Parameters such as voltage and frequency were determined for the optimum circular scan pattern and a look‐up‐table was stored to be later used for reconstruction of a full 3D OCT C‐Scan. In case the OCT data was further evaluated for angiography, the variance of consecutive intensity tomograms (circles) was calculated to depict motion contrast and to visualize the dynamic blood flow. In addition, a smaller pitch between circular B‐scans was necessary to detect small signal fluctuations, and a slower frame rate of 0.1 Hz (resulting in 1800 B‐scans in 5 seconds with averaged pitch of ~0.35 μm) was chosen. For illustration of an OCT and OCTA scan pattern, see Supporting Information, Figure S1. According to the Nyquist theorem sufficient sampling along the individual circles was only fulfilled in the inner part of the spiral up to a FOV diameter of ~900 μm. However, no noticeable image degradation was observed.

## 3 RESULTS AND DISCUSSION

**Figure 3:**
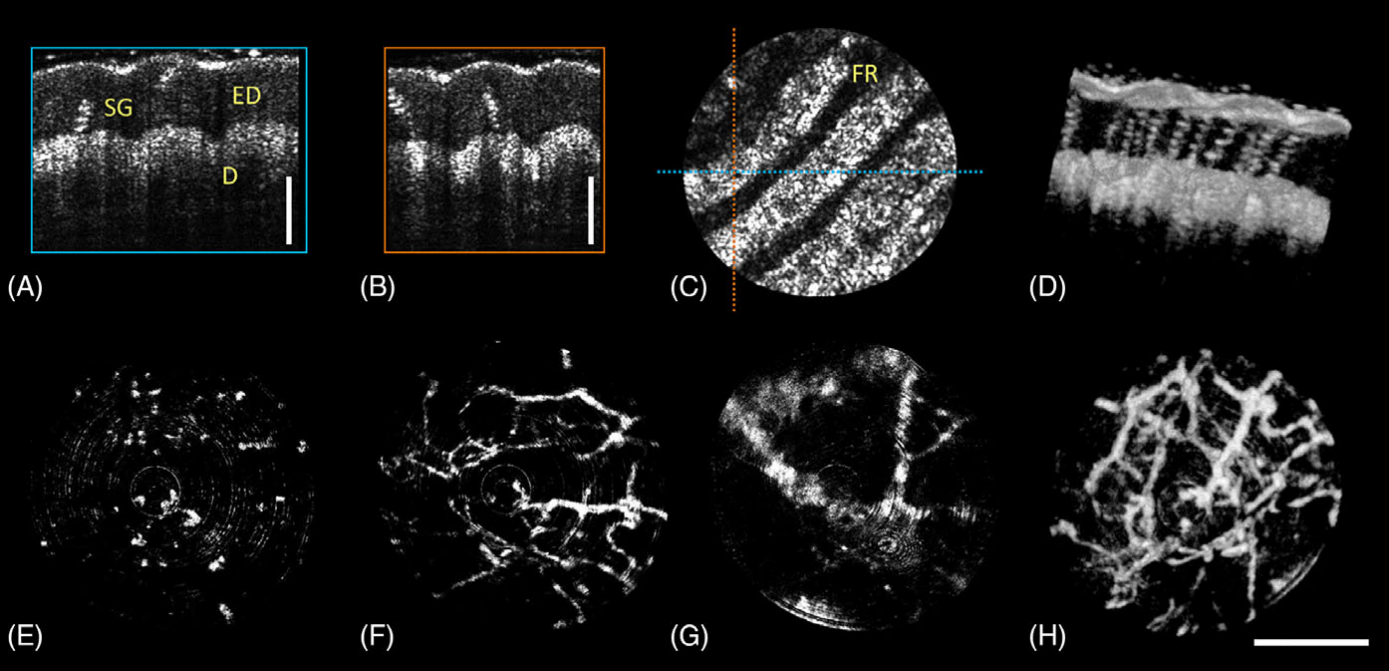
OCT and OCTA images acquired with the piezo endoscope. (A)‐(D) OCT images of a fingertip where the dermis (D), epidermis (ED), friction ridges (FR) and sweat glands (SG) can be identified in the cross‐sectional images (A), (B) the en‐face view (C) and the rendered 3D volume (D). (E)‐(H) OCTA images of the human palm at different depth within the tissue: (E) ~230‐350 μm, (F) ~360‐680 μm, (G) ~880‐1170 μm, (H) 3D representation of the data. Scale bar: 500 μm

Figure 3 shows OCT images acquired from the fingertip and OCTA images from the palm. Informed consent from the healthy volunteer was obtained prior to the measurements, and the study was in agreement with the tenets of the Declaration of Helsinki and approved by the institutional ethics committee. Figure 3A‐D shows intensity OCT images of a human fingertip as cross‐sections in all three dimensions as well as a rendered 3D volume with a FOV of 1.16 mm (max. voltage amplitude 42 V). The friction ridges, the dermis and epidermis as well as sweat glands are clearly visible. The penetration depth is limited due to the very short focal distance and limited DOF inside the tissue. Figure 3E‐H show OCTA images of the palm with a FOV of 1.26 mm (max. voltage amplitude 44.5 V). These images were generated by an en‐face projection of multiple slices over a certain depth. Small capillary vessel loops that are vertically oriented in the very top layer (~230–350 μm depth in tissue), smaller vessels at a depth of ~360–680 μm, and also larger ones (depth of ~880–1170 μm) are clearly visible. A visualization of the 3D representation of the finger and palm can be found in Figures S2 and S3, respectively.

In this paper, we present a short forward imaging piezo endoscope head design. It can be inserted into the working channel of a rigid standard‐of‐care endoscope or, if further miniaturized in terms of its diameter (by changing to a different housing and smaller lens), be used with a flexible endoscope. If configured to operate in non‐contact mode at a working distance of up to 5 mm, the rigid length of the probe can further be reduced to 6.7 mm by eliminating the objective lens and terminating the probe at the distal end by a simple cover glass. The acquisition time of presented OCTA data‐sets is currently 5 seconds—similar to the time that it takes to perform retinal OCTA measurements. Since the probe works in contact mode, it is possible to remain stable and avoid significant motion during the acquisition. By decreasing the sampling in between individual spirals and consequently the acquisition time, it was also possible to visualize vasculature—however—at reduced motion contrast. The acquisition time can be further shortened by increasing the resonance frequency of the fiber scanner. However, this would lead to a degradation of image quality due to significant undersampling, which could only be avoided by using a faster laser (eg, FDML Laser from ref. 15).

## 4 CONCLUSION

In conclusion, a forward imaging endoscope to perform OCT as well as OCTA imaging was presented. By incorporating a plano‐convex lens, a short rigid probe length of 11.3 mm with a transversal resolution of 12 μm and FOV of 1.3 mm is achieved. The endoscope operates in contact mode to minimize motion artifacts, which is particularly important for the highly motion sensitive acquisition of OCTA images and consequently allowed a 3D visualization of small vessels successfully. Future probe designs may further reduce the overall diameter to ~ 2.2 mm to allow feasibility tests with a flexible endoscope, for example for early diagnosis of bladder cancer.

## Supporting information


**Figure S1.** Look up table of spiral scan pattern that was generated before the measurement was performed to reconstruct the 3D image, (A) Look up table with 360 concentric circles generated for OCT measurement, (B) Look up table with 1800 concentric circles, created for OCTA measurements. Zoom in region to illustrate the much smaller pitch between spirals for the OCTA measurement. Slight asymmetry in the circular pattern is possibly caused by asymmetric behavior of the two axis of the piezo tube that was not perfectly corrected during the calibration procedure. Scale bar: 100 μm
**Figure S2.** 3D volume of OCT finger tip
**Figure S3.** 3D volume of OCT angiogram of palm
