# Supplementary material for: Endoscopic optical coherence tomography angiography using a forward imaging piezo scanner probe
Source: J Biophotonics. 2019 Jan 30;12(4):e201800382. doi: 10.1002/jbio.201800382 (PMC7065608; doi:10.1002/jbio.201800382)

Figure S1:

Figure Caption:
Look up table of spiral scan pattern that was generated before the measurement was performed in order to reconstruct the 3D image, a) Look up table with 360 concentric circles generated for OCT measurement, b) Look up table with 1800 concentric circles, created for OCTA measurements. Zoom in region to illustrate the much smaller pitch between spirals for the OCTA measurement. Slight asymmetry in the circular pattern is possibly caused by asymmetric behavior of the two axis of the piezo tube that was not perfectly corrected during the calibration procedure. Scale bar: 100µm


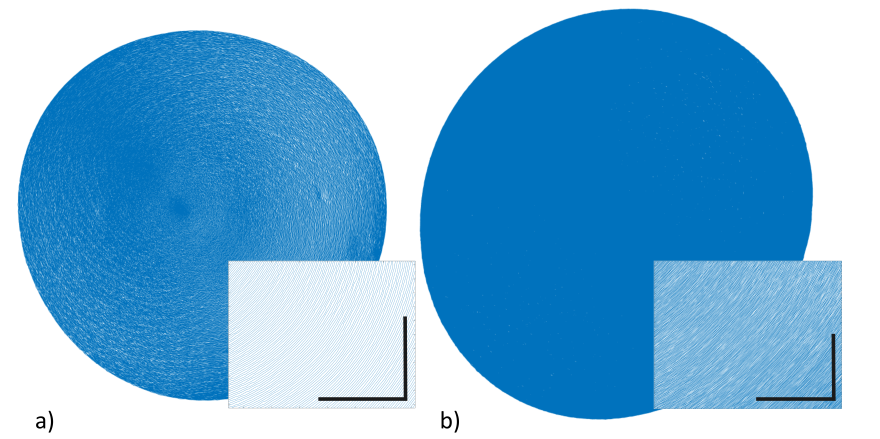


Figure S2:
Figure Caption: 3D volume of OCT finger tip


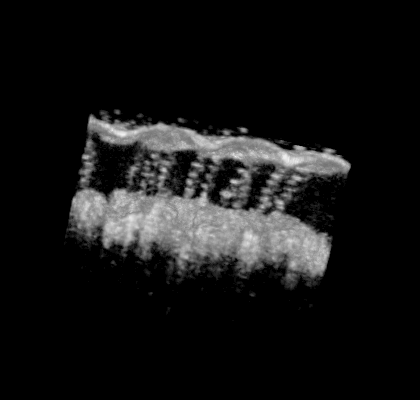


Figure S3:
Figure Caption: 3D volume of OCT angiogram of palm


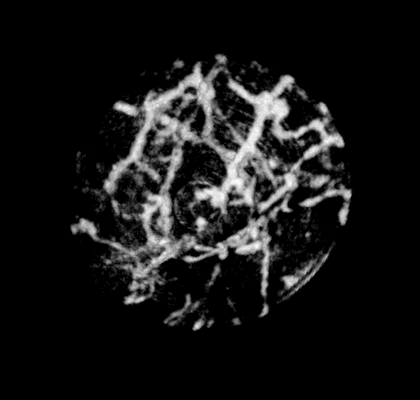

Supplement: Supplementary file 1 — Figure S1. Look up table of spiral scan pattern that was generated before the measurement was performed to reconstruct the 3D image, (A) Look up table with 360 concentric circles generated for OCT measurement, (B) Look up table with 1800 concentric circles, created for OCTA measurements. Zoom in region to illustrate the much smaller pitch between spirals for the OCTA measurement. Slight asymmetry in the circular pattern is possibly caused by asymmetric behavior of the two axis of the piezo tube that was not perfectly corrected during the calibration procedure. Scale bar: 100 μm Figure S2. 3D volume of OCT finger tip Figure S3. 3D volume of OCT angiogram of palm [file JBIO-12-e201800382-s001.docx]
